# Dermoscopy in the Evaluation of Tinea Corporis: A Potential to Optimize Diagnostic Accuracy

**DOI:** 10.7759/cureus.114320

**Published:** 2026-08-11

**Authors:** Yamunaraj S D, Rahul H V, Chethan Kumar, Jayaprakash M H, Sagarika B, Jithin Jayaraj

**Affiliations:** 1 Department of Dermatology, Mandya Institute of Medical Sciences, Mandya, IND; 2 Department of Dermatology, Adichunchanagiri Institute of Medical Sciences, Adichunchanagiri University, Bellur, IND; 3 Department of Dermatology, Siddaganga Medical College and Research Institute, Tumkur, IND; 4 Department of Surgery, Jawaharlal Nehru Medical College, Belagavi, IND

**Keywords:** dermatophytes, dermoscopy, dermoscopy image analysis, dermoscopy of dermatophytosis, fungal culture, fungal infection, koh mount, non-invasive diagnosis and management, skin scaling, tinea corporis

## Abstract

Introduction

Tinea corporis, or ringworm, is a fungal infection caused by dermatophytes that affects the skin. It is a significant public health concern, particularly in tropical regions like India, due to issues like antifungal resistance and improper use of combination creams. Traditional diagnostic methods, such as potassium hydroxide (KOH) tests and fungal cultures, are reliable but time-consuming and require laboratory access. Dermoscopy, a non-invasive diagnostic tool, offers a faster alternative by revealing distinct patterns in skin infections, including tinea corporis, invisible to the naked eye.

Objective

This study aimed to analyse the clinical profile of tinea corporis and describe the utility of dermoscopy as an adjunctive diagnostic tool.

Methods

A prospective cross-sectional observational study at a tertiary care hospital in Karnataka, conducted from April to August 2024, included 200 clinically confirmed tinea corporis cases. Patients were evaluated using KOH tests and dermoscopy. Dermoscopic findings, recorded with a Derma India® M10A30 hybrid dermatoscope, were analyzed for specific patterns like scales and background color changes to aid diagnosis.

Results

The study indicated that 61% of the cases were men, with the highest prevalence observed in individuals aged 31-40 years. The key contributing risk factors were identified as poor hygiene and a previous history of dermatophytosis, both of which significantly increased susceptibility to infection. Dermoscopic examination revealed distinctive features, including peripheral scaling, reddish-brown backgrounds, and black dots, which were commonly present and proved essential for the precise and reliable diagnosis of tinea corporis.

Conclusion

Dermoscopy is a valuable, rapid, and non-invasive adjunctive diagnostic tool that complements clinical examination and potassium hydroxide microscopy. It facilitates the recognition of characteristic dermoscopic features, helping clinicians differentiate tinea corporis from other skin conditions, especially in resource-constrained settings.

## Introduction

Tinea corporis, also known as ringworm of the body, is a superficial fungal infection that affects the skin, caused by dermatophytes. These fungi colonize keratinized tissues such as the skin, hair, and nails [1]. Over recent years, dermatophytosis has become a significant public health challenge, especially in tropical and subtropical regions like India [2]. Factors such as antifungal resistance, irrational use of over-the-counter antifungal-steroid combination creams, and environmental conditions have contributed to the increased prevalence of this condition [3]. Traditionally, the diagnosis of tinea corporis relies on clinical features, microscopic examination of skin scrapings using potassium hydroxide (KOH), and fungal culture [4]. While effective, these conventional methods are time-consuming, require laboratory infrastructure, and depend on the expertise of trained personnel, limiting their accessibility in resource-poor settings [5].

In this context, dermoscopy has emerged as a promising diagnostic tool that enhances the evaluation of various dermatological conditions, including tinea corporis [6]. Dermoscopy, also known as epiluminescence microscopy or skin surface microscopy, is a non-invasive technique that allows for magnified visualization of the skin's surface, revealing patterns and structures not visible to the naked eye [7]. Initially popularized in the diagnosis of pigmented lesions and skin cancers, dermoscopy has since expanded to encompass inflammatory and infectious skin diseases, including fungal infections [8]. In the case of tinea corporis, dermoscopy enables clinicians to identify specific features associated with the infection, such as the presence of diffuse scales, peripheral scaling, moth-eaten borders, and globular patterns, which help in differentiating it from other skin conditions [9].

The use of dermoscopy in the diagnosis of tinea corporis holds several advantages. First, it is a simple, rapid, and non-invasive method that can be performed at the bedside without the need for specialized laboratory tools [10]. This is particularly useful in busy outpatient settings or remote areas where access to advanced diagnostic facilities may be limited [11]. Second, dermoscopy facilitates the documentation of skin lesions through digital imaging, which allows for better tracking of disease progression and response to treatment [12]. Moreover, by improving diagnostic accuracy, dermoscopy helps in reducing unnecessary use of antifungals and preventing misdiagnosis, which can lead to inappropriate treatments and increased drug resistance [13].

The utility of dermoscopy in distinguishing tinea corporis from other skin conditions, such as eczema or psoriasis, which may present with similar clinical features [14]. Typical dermoscopic findings in tinea corporis include a reddish-brown or greyish-black background, white scales, and the presence of black dots or globules representing the broken hair shafts [15]. Additionally, patterns of scaling such as outward peeling or scales prominent over skin creases can provide clues to the stage of infection [16].

Dermoscopy is a valuable diagnostic tool in the evaluation of tinea corporis, offering a fast, accessible, and accurate method for optimizing diagnostic accuracy [17]. Its non-invasive nature and ability to provide real-time insights make it an essential addition to the clinician’s toolkit for managing dermatophytosis, especially in settings with limited laboratory resources [18].

The study aims to evaluate the clinical profile of tinea corporis, focusing on its presentation, demographic patterns, and associated risk factors. By examining a cohort of confirmed cases, the research seeks to provide a comprehensive understanding of the disease's prevalence across different age groups, gender distributions, and comorbidities. Additionally, the study aims to describe specific dermoscopic findings, including patterns of scaling, background color variations, and the presence of dots and globules. These observations may assist clinicians in the clinical evaluation of patients with tinea corporis.

## Materials and methods

This prospective cross-sectional observational study was conducted in the Department of Dermatology, J.J.M. Medical College and Hospital, Davangere, Karnataka, India, from April 2024 to August 2024, after obtaining approval from the Institutional Ethics Committee (IEC). The study was carried out at the institute where the ethical approval was granted. Written informed consent was obtained from all the participants before enrollment.

A total of 200 consecutive patients with clinically suspected tinea corporis attending the dermatology outpatient department were recruited using consecutive sampling. The sample size was determined based on the expected prevalence of characteristic dermoscopic findings reported in previous studies, considering a 95% confidence interval and adequate statistical power.

The inclusion criteria were patients of either sex aged ≥18 years with clinically suspected tinea corporis, potassium hydroxide (10% KOH) mount-positive cases, and patients willing to provide written informed consent.

The exclusion criteria were patients who had received topical or systemic antifungal therapy or topical corticosteroids within the preceding four weeks, KOH-negative lesions, patients with mixed dermatophytic infections in whom dermoscopic interpretation was difficult, and patients unwilling to participate.

For each participant, demographic details, duration of the disease, presenting symptoms, personal hygiene practices, history of previous dermatophytosis, family history, associated comorbidities, prior treatment, and detailed cutaneous examination findings were recorded using a structured proforma.

Skin scrapings were collected from the active peripheral margin of representative lesions and examined using 10% potassium hydroxide (KOH) preparation for the confirmation of dermatophyte infection. Dermoscopic evaluation was subsequently performed in all KOH-positive patients using a Derma India® M10A30 hybrid dermatoscope (DermaIndia, Chennai, India). Clinical and dermoscopic images were captured using a Samsung Galaxy A34 smartphone coupled to the dermatoscope using the manufacturer's standard smartphone attachment. Dermoscopic examinations and image acquisition were performed by two investigators using a standardized imaging protocol. The dermoscopic findings were subsequently reviewed by three investigators experienced in dermoscopy, and the final observations were recorded by consensus using predefined dermoscopic criteria.

Dermoscopic examination evaluated the presence of background colour changes (pale red, reddish-brown and greyish-black), diffuse and peripheral scaling, scales over skin creases, outward peeling, moth-eaten scales, reddish-brown dots and globules, black dots and globules, broken hairs, follicular micropustules, dotted vessels and crusts.

The potential confounding factors, including age, sex, diabetes mellitus, previous dermatophytosis, family history, personal hygiene practices, sharing of clothes, seasonal exacerbation, and prior medication use, were documented and considered during the interpretation of the findings.

Statistical analysis

Data were entered into Microsoft Excel (Microsoft, Redmond, WA) and analysed using IBM SPSS Statistics, version 23 (IBM Corp., Armonk, NY, USA). Categorical variables were expressed as frequencies and percentages, while continuous variables were presented as mean±standard deviation (SD). Associations between categorical variables were assessed using the chi-square test or Fisher's exact test, as appropriate. A p-value <0.05 was considered statistically significant.

## Results

Table 1 presents the distribution of 200 individuals across different age groups, with a breakdown by gender. Men constitute 61% (122 individuals) and women represent 39% (78 individuals). The largest group is aged 31-40 years, making up 25.5% of the total population. Age groups 21-30 and 41-50 years also show significant numbers, with 40 and 38 individuals, respectively. The distribution decreases steadily in older age groups, with minimal representation in the 81- to 90-year range.

**Table 1:** Comparison of different age groups among gender

Age group	Male	Female	Total
0-10	1	-	1
11-20	11	4	15
21-30	26	14	40
31-40	31	20	51 (25.5%)
41-50	20	18	38
51-60	18	13	31
61-70	9	6	15
71-80	4	3	7
81-90	2	-	2
Total	122 (61%)	78 (39%)	200

Table 2 outlines various risk factors and their associated frequencies. The most common risk factors are not bathing daily (33.5%) and family history of similar complaints (33%). A past history of dermatophytosis contributes to the extent of 31.5%, while seasonal exacerbation and sharing of clothes contribute 20% and 17.5%, respectively. Diabetes mellitus is a risk factor for 18% of the individuals.

**Table 2:** Risk factors

Risk Factor	Frequency
Family history of similar complaints	66 (33%)
Sharing of clothes	35 (17.5%)
Not bathing daily	67 (33.5%)
Past history of dermatophytosis	63 (31.5%)
Seasonal exacerbation	40 (20%)
Diabetes mellitus	36 (18%)

Table 3 shows additional sites involved in cases of tinea corporis. Tinea cruris is the most commonly associated condition, appearing in 132 cases. Onychomycosis is the second most frequent, occurring in 61 cases. Tinea faceii and tinea mannum are less common, with 13 and one reported cases, respectively, indicating a wide range of secondary site involvement in tinea corporis cases.

**Table 3:** Other sites involved along with tinea corporis

Other Sites Involved Along with Tinea Corporis	Frequency
Tinea cruris	132
Onychomycosis	61
Tinea faceii	13
Tinea mannum	1

**Figure 1:**
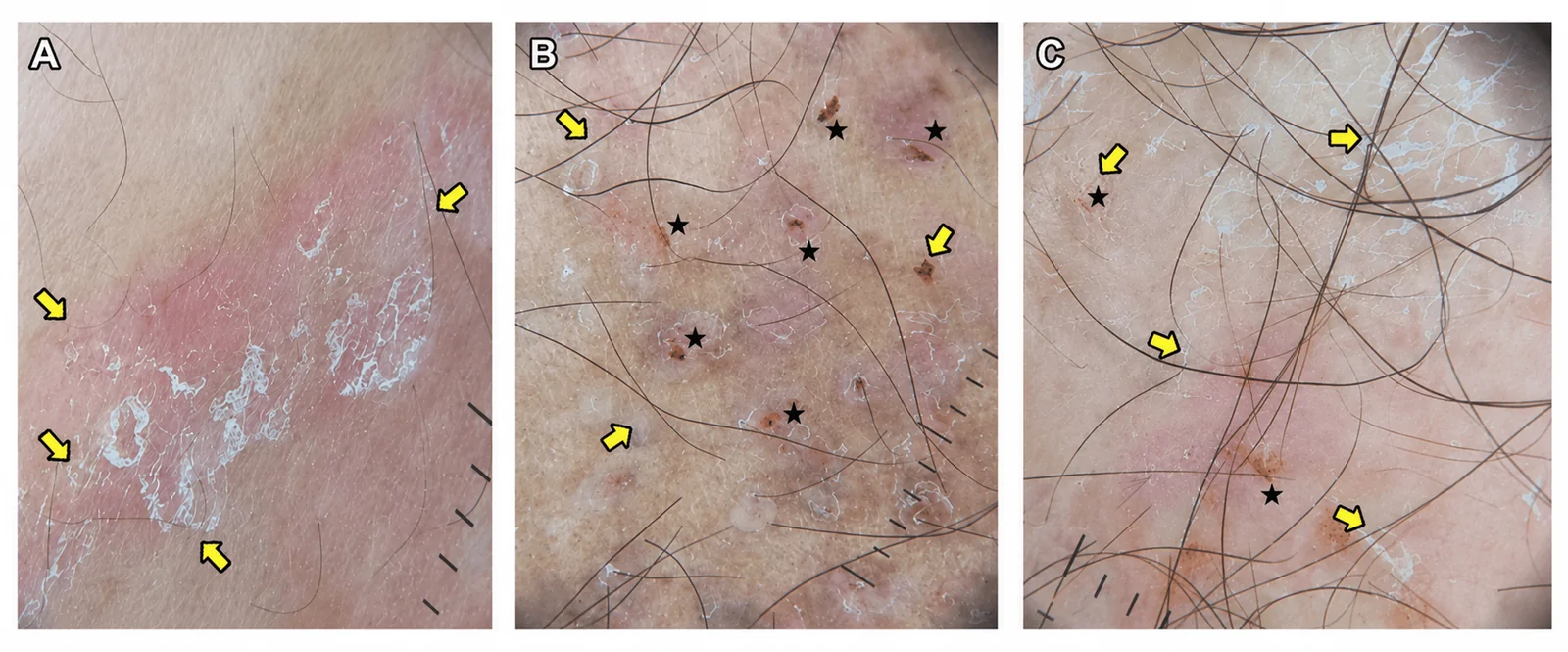
Early dermoscopic features of tinea corporis (A) Peripheral white scales (yellow arrows) over a pale erythematous background. (B) Reddish-brown dots and globules (black stars) with superficial white scales (yellow arrows) on a pale erythematous background. (C) Early peripheral white scales (yellow arrows) with focal reddish-brown pigmentation (black stars) on a subtly erythematous background.

**Figure 2:**
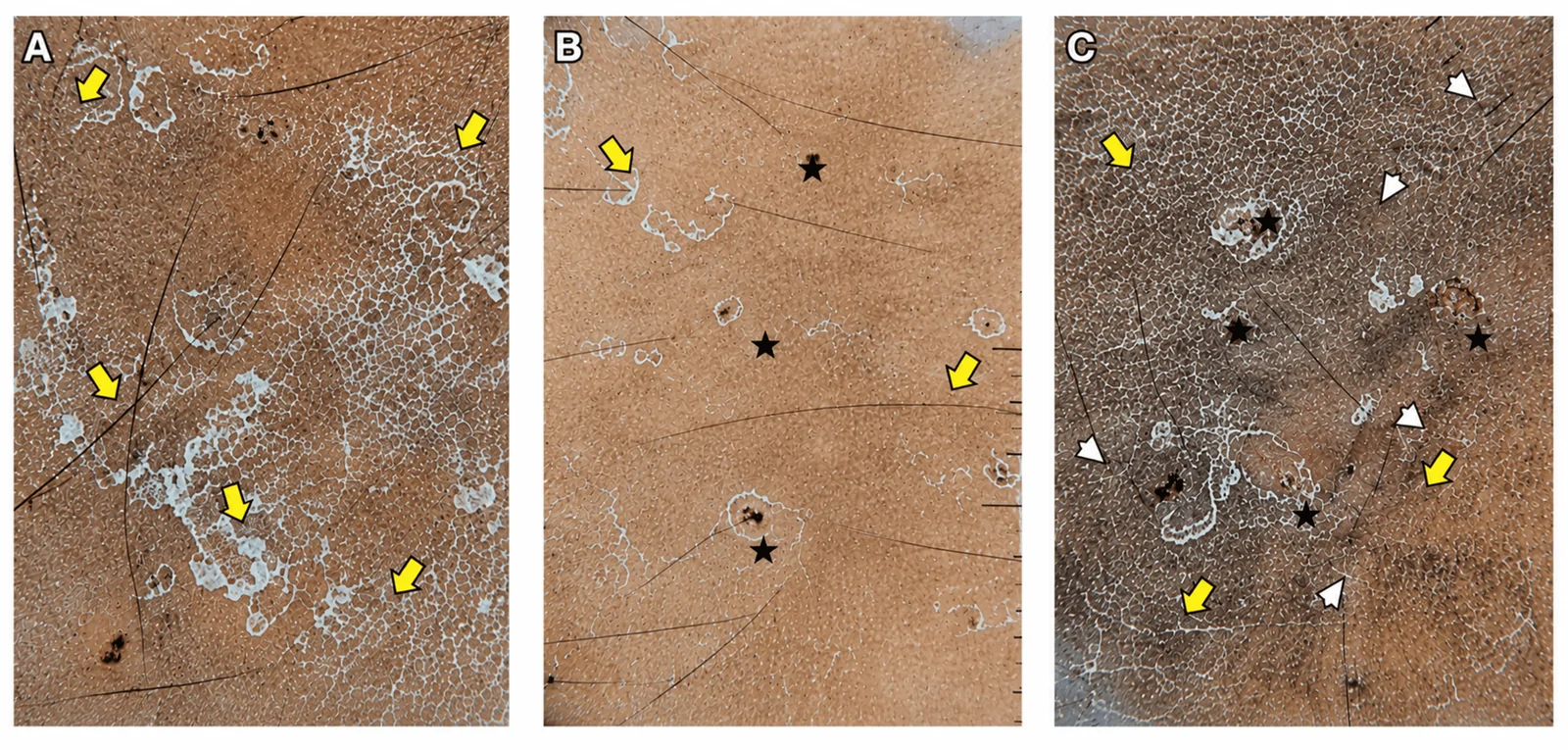
Advanced dermoscopic features of tinea corporis (A) Prominent diffuse and peripheral white scales (yellow arrows) with outward peeling (collarette) and moth-eaten scaling. (B) Greyish-brown background with peripheral white scales (yellow arrows) and scattered black dots/globules (black stars). (C) Greyish-black background showing black dots/globules (black stars), diffuse white scales (yellow arrows), and broken hairs (white arrowheads), features consistent with advanced tinea corporis.

Table 4 summarizes the dermoscopic findings with their frequencies. Peripheral scales (183), outward peeling of scales and moth-eaten scales (172 and 151), and diffuse scales (161) were the most common findings. Pale red to reddish-brown background (130), reddish-brown dots and globules (122), and black dots and globules (109) were also frequently observed. Less common findings included crusts (23), dotted vessels (5), and follicular micropustules (2). Representative dermoscopic images demonstrating the early features of tinea corporis, including peripheral scaling, superficial scales, and reddish-brown dots and globules, are shown in Figure 1, whereas Figure 2 illustrates advanced dermoscopic changes characterized by diffuse and peripheral scaling, outward peeling, moth-eaten scales, greyish-black background, black dots/globules, and broken hairs.

**Table 4:** Dermoscopy findings and frequency

Dermoscopy Findings	Frequency
Pale red to reddish brown background	130
Reddish brown dots and globules	122
Greyish black background	98
Black dots and globules	109
Diffuse scales	161
Peripheral scales	183
Scales prominent over the skin creases	164
Outward peeling of scales & moth-eaten scales	172 & 151
Crusts	23
Follicular micropustules	2
Dotted vessels	5
Broken hairs	36

Most patients were men in their 40s. Tinea cruris was the most common association with tinea corporis, followed by onychomycosis and tinea faceii. Early dermoscopic findings included dotted vessels, reddish-brown dots on a reddish-brown background, and superficial white scales. In later cases, black dots on a greyish-black background with whitish scales appeared. Additional findings included outward peeling, moth-eaten and diffuse scaling, scales over skin creases, micropustules, and broken hairs.

## Discussion

Tinea corporis remains one of the most common superficial dermatophyte infections worldwide and continues to pose a significant public health challenge because of increasing antifungal resistance, widespread misuse of topical corticosteroid-antifungal combinations, and frequent clinical overlap with other inflammatory dermatoses [19]. Although KOH microscopy and fungal culture remain the conventional diagnostic methods, they require laboratory infrastructure and may delay diagnosis [4,5]. Dermoscopy has emerged as a rapid, non-invasive adjunct that enables the visualization of characteristic morphological features, thereby improving bedside diagnosis and facilitating differentiation from clinically similar disorders such as psoriasis and eczema [6-18].

The present study demonstrated that peripheral scales, diffuse scales, scales over skin creases, and outward peeling were the predominant dermoscopic findings, while reddish-brown and greyish-black backgrounds with corresponding dots and globules represented characteristic pigmentary changes. Less frequent findings included crusts, dotted vessels, follicular micropustules, and broken hairs, suggesting a spectrum of dermoscopic features reflecting different stages of disease progression.

Our findings closely parallel those of Ankad et al. [20], who reported white scales together with brown-to-black dots and globules as the hallmark dermoscopic findings of dermatophytosis. They further observed that reddish-brown dots and dotted vessels predominated in early lesions, whereas darker brown-to-black globules became increasingly prominent with increasing disease duration. Likewise, our study demonstrated a transition from reddish-brown backgrounds with reddish-brown dots to greyish-black backgrounds with black dots and globules, indicating progressive dermoscopic evolution.

Similarly, Bhat et al. [21] described erythema, follicular micropustules, brown spots surrounded by whitish-yellow halos, and characteristic hair changes including broken, wavy, and Morse code hairs. Although follicular micropustules were infrequent in our series, broken hairs and follicular involvement were identified, supporting the usefulness of dermoscopy in detecting follicular extension that may not be clinically evident.

The predominance of peripheral scaling, outward peeling, and moth-eaten scales in our study is in agreement with the review by Lim et al. [22], who identified these as among the most reliable dermoscopic features of cutaneous fungal infections. They also emphasized diffuse erythema, dotted vessels, broken hairs, and follicular micropustules as additional supportive findings, many of which were observed in our patients, albeit with lower frequencies.

Our observations are also consistent with those of Gadekar et al. [23], who demonstrated that perifollicular scales, follicular micropustules, yellow hairs, transparent hairs, and Morse code hairs are indicative of follicular involvement, particularly in steroid-modified dermatophytosis. Although advanced hair abnormalities were less frequent in our cohort, the presence of broken hairs and follicular changes supports dermoscopy as a useful tool for assessing disease severity and possible follicular invasion.

The characteristic scaling patterns observed in our study are also supported by previous descriptions of dermoscopy in inflammatory and infectious dermatoses by Errichetti and Stinco [6] and Micali and Lacarrubba [9], who emphasized that peripheral scaling, pigmentary alterations, and follicular changes are reproducible features useful for differentiating superficial fungal infections from conditions such as psoriasis and eczema. Similarly, Liu and Zou [17] highlighted the expanding role of dermoscopy beyond pigmented lesions into general dermatology, while Heckler et al. [18] stressed the need for rapid, accurate bedside diagnostic techniques for dermatomycoses to facilitate early treatment and improve patient outcomes.

Overall, our findings corroborate previous literature and further demonstrate that dermoscopy provides a rapid recognition of characteristic features such as peripheral scales, outward peeling, diffuse scales, pigmentary background changes, dots and globules, and hair abnormalities, making it an effective adjunct to clinical examination and KOH microscopy for the diagnosis of tinea corporis [6-23].

Limitations

This was a single-centre study conducted in a tertiary care hospital and included only KOH-positive patients, which may limit the generalizability of the findings. Fungal culture and species identification were not performed; therefore, dermoscopic patterns could not be correlated with individual dermatophyte species. In addition, no comparison group of clinically similar dermatoses was included; therefore, the diagnostic performance of dermoscopy in differentiating tinea corporis from other conditions could not be assessed. Larger multicentric studies with culture confirmation are required to validate these findings.

## Conclusions

Dermoscopy is a valuable adjunctive diagnostic tool that offers a fast, cost-effective, and non-invasive method for diagnosing cutaneous fungal infections. Its ability to provide detailed visualization of skin structures allows clinicians to detect subtle changes associated with fungal infections, such as specific patterns of scaling, pigmentation, and vascular alterations. By facilitating the recognition of characteristic dermoscopic features, dermoscopy reduces the need for more invasive procedures like biopsies and can lead to earlier detection and treatment. This makes it a practical and accessible option in clinical settings, improving patient outcomes and streamlining the diagnostic process for skin infections.
